# *Nicandra physalodes* (Linn.) Gaertn. Polysaccharide-Stabilized Peanut Oil Body Composite Emulsion Gels: Spontaneous Gelation Behavior, Rheological Properties, and 3D Printing Performance

**DOI:** 10.3390/foods15162921

**Published:** 2026-08-20

**Authors:** Siqi Sun, Fusheng Chen, Dingyang Lv

**Affiliations:** College of Food Science and Technology, Henan University of Technology, Zhengzhou 450001, China; siqisun1130@163.com (S.S.); dingyanglv@163.com (D.L.)

**Keywords:** *Nicandra physalodes* (Linn.) Gaertn. polysaccharide, peanut oil body, emulsion gels, rheological property, 3D printing

## Abstract

In this study, peanut oil bodies were stabilized using different concentrations of *Nicandra physalodes* (Linn.) Gaertn. polysaccharide (NPGP) to fabricate composite emulsion gels. The physical stability, rheological properties, and 3D printing performance of the resulting gels were systematically investigated. The results revealed that the composite systems were capable of self-gelation when the NPGP concentration exceeded 1.0 wt%. Furthermore, the gel hardness and physical stability were improved with the elevation of NPGP concentration. Within the linear viscoelastic region, the samples exhibited dominant elastic responses and maintained solid-like properties throughout the entire test period (G′ > G″). Further 3D printing tests demonstrated that the composite systems possessed favorable formability, with printing precision and printing stability reaching 98.86 ± 0.14% and 97.11 ± 0.07%, respectively. Collectively, as a promising commercial pectin-like polysaccharide resource, NPGP can effectively enhance the stability of peanut oil body emulsions. This study also provides novel strategies and theoretical foundations for the structural regulation and functional utilization of oil bodies.

## 1. Introduction

Oil body emulsions are a type of safe, green, and naturally oil-in-water system that does not require additional emulsification processes. They also possess excellent biocompatibility, degradability, and nutritional value, and show unique advantages and application potential in areas such as fat substitution, drug and nutrient delivery, and the construction of green functional materials [1,2,3]. Nevertheless, oil bodies are prone to destabilization during processing and storage, manifested mainly as droplet aggregation, creaming, flocculation and even demulsification, which greatly hinders their practical utilization [4]. Accordingly, it is of great significance to develop efficient and mild stabilization strategies that can preserve the intrinsic structure of these oil bodies.

At present, researchers commonly utilize exogenous additives to stabilize oil bodies [5]. Among these additives, polysaccharides have attracted extensive attention owing to their abundant sources, low cost, favorable biocompatibility, low toxicity [6], and outstanding thickening and stabilizing performance. Existing studies have demonstrated that polysaccharides including pectin, gum arabic, alginate, sodium carboxymethyl cellulose, xanthan gum and chitosan can improve the stability of oil bodies via distinct interaction mechanisms [7]. For instance, in peanut oil body systems, emulsion gels formed by incubating xanthan gum-modified peanut oil body emulsions with gallic acid in a water bath at 50 °C for 2 h exhibit superior physical properties and enhanced mechanical strength [8]. Adjusting the pH to 4.5 facilitates the adsorption of sodium alginate onto soybean oil bodies, which strengthens electrostatic and steric repulsion between oil bodies while weakening van der Waals attraction, thereby endowing the system with favorable anti-aggregation capacity and emulsifying stability [9]. Electrostatic deposition technology was adopted to coat curcumin-loaded camellia oil bodies with gum arabic and chitosan. This strategy not only provides effective protection for encapsulated curcumin, but also preserves the intrinsic structural integrity of oil bodies, maintaining the structural stability and functional properties of oil body-based emulsion systems [10]. The above polysaccharide-stabilized systems differ in their core oil body-stabilizing mechanisms depending on preparation approaches, which further leads to discrepancies in their applicable scenarios [11,12].

Nevertheless, the aforementioned stabilization strategies for oil bodies generally rely on auxiliary external conditions such as pH adjustment, ion addition, and temperature variation. Such treatments may alter the native interfacial structure and physicochemical properties of oil bodies [13,14,15], and even impair their inherent natural properties, thus restricting their practical application. In addition, the implementation of these treatments requires additional cost investment. Accordingly, researchers are pursuing simpler techniques and methodologies for oil body stabilization. Recent studies have identified a series of gel-forming polysaccharides derived from traditional plants [16] which are capable of spontaneous gelation without induction by exogenous stimuli. Moreover, these polysaccharides can form composite gels with plant proteins via non-covalent interactions [17]. The oil body interface is normally stabilized by interfacial proteins. Accordingly, it is hypothesized that these self-gelling polysaccharides can interact with interfacial proteins on oil bodies to stabilize oil body emulsions.

*Nicandra physalodes* is an erect annual herb belonging to the Solanaceae family. The seeds in its berries are its main by-product, and the outer seed coat contains a rich layer of pectin polysaccharides. After sufficient seed soaking, *Nicandra physalodes* seed polysaccharide (NPGP) can be dissolved in water via manual rubbing [18]. As a typical self-gelling polysaccharide, NPGP is capable of spontaneous gel formation at ambient temperature. Existing studies have demonstrated that NPGP exhibits superior emulsification and emulsion stabilization capacities compared with apple pectin and citrus pectin, which can be attributed to its lower interfacial tension, higher system viscosity, more uniform droplet size distribution, stronger electrostatic repulsion, and thicker interfacial layers [19]. Nevertheless, oil bodies possess unique native interfacial structures, and their interactions with NPGP together with the corresponding stabilization mechanism remain to be further verified. Notably, if NPGP can form an emulsion gel with a certain network structure on the basis of stabilizing peanut oil body emulsions, the system is expected to achieve improved shape fidelity and structural retention, making it a promising edible 3D printing ink. In recent years, emulsion gel-based 3D printing technology has advanced rapidly in the food sector, demonstrating significant promise, particularly in the formulation of diets for people with dysphagia, the provision of personalized nutrition for patients with swallowing disorders [20], and plant-based meat alternatives that simulate animal fat [21].

Accordingly, this study aims to elucidate the stabilization mechanism of peanut oil body emulsions by NPGP and to evaluate the feasibility of applying the resulting composite emulsion gels in food 3D printing. Specifically, this work investigated the effects of NPGP concentration on the structure and rheological properties of peanut oil body emulsions, as well as on their printability and structural retention during 3D printing. The results can provide a theoretical basis and data support for the application of NPGP in oil body stabilization and the development of novel plant-based food 3D printing inks.

## 2. Materials and Methods

### 2.1. Materials and Chemicals

*Nicandra physalodes* seeds were purchased from Mengzi City, Honghe Hani and Yi Autonomous Prefecture, Yunnan Province, and stored at −20 °C. Peanut kernels (YuHua 37 cultivar) were obtained from the Henan Academy of Agricultural Sciences (Zhengzhou, China) and stored at 4 °C. Anhydrous ethanol was purchased from Tianjin Tianli Chemical Reagent Co., Ltd. (Tianjin, China). Analytical-grade chemicals and solvents were obtained from Shanghai Macklin Biochemical (Shanghai, China).

### 2.2. Extraction of Polysaccharides

The polysaccharide extraction procedure was performed in line with the method reported by Guo et al., with minor modifications [18]. Briefly, approximately 5.0 g of *Nicandra physalodes* seeds were wrapped in 400-mesh fine nylon gauze and manually squeezed and kneaded in 150 mL of deionized water for 10 min. The polysaccharide dispersion was precipitated overnight at 4 °C by adding three volumes of 95% (*v*/*v*) ethanol. The precipitate was harvested and redissolved in deionized water, followed by rotary evaporation to eliminate residual ethanol. The resulting mixture was lyophilized, designated as NPGP, and reserved for subsequent characterizations.

### 2.3. Extraction of Peanut Oil Bodies

Peanut oil bodies were extracted following the procedure of Wang et al. [22]. Dehulled peanut kernels were soaked in deionized water at a solid-to-liquid ratio of 1:7 (*w*/*v*) for 18 h at 4 °C. The soaked peanuts were homogenized for 2 min using a multifunctional food processor (Model C022E, Joyoung Co., Ltd., Jinan, China). The resulting slurry was filtered through four layers of filter cloth to remove solid residues, and the obtained filtrate was centrifuged at 5000 rpm for 20 min at ambient temperature to harvest the upper crude peanut oil body layer. Subsequently, the crude oil bodies were washed twice with deionized water at a ratio of 1:4 (*w*/*v*), followed by another centrifugation step at 5000 rpm for 30 min under ambient temperature; the top concentrated fraction was finally collected as purified peanut oil bodies.

### 2.4. Proximate Composition Analysis

The moisture and ash contents of samples were determined in accordance with the direct drying method (GB 5009.3-2016 [23]) and high-temperature ignition method (GB 5009.4-2016 [24]), respectively, while the protein content was quantified via the Kjeldahl method (GB 5009.5-2016 [25]). The galacturonic acid content of polysaccharides was measured using the carbazole–sulfuric acid colorimetric method. The molybdenum blue colorimetric method (GB/T 5537-2008 [26]) and chloroform–methanol extraction method were separately adopted to quantify phospholipid and total lipid contents in peanut oil bodies.

### 2.5. Preparation of NPGP–Peanut Oil Body Composite Emulsion Gels

Peanut oil bodies were dispersed in ultrapure water at a mass ratio of 1:2 under continuous stirring for 5 h to obtain pristine peanut oil body emulsions. NPGP powder was incorporated into the above oil body aqueous dispersions to reach final polysaccharide concentrations of 0.5 wt%, 1.0 wt%, 1.5 wt%, 2.0 wt% and 2.5 wt%. After homogeneous manual stirring, each mixture was sheared using a high-speed homogenizer at 10,000 rpm for 3 min, and composite systems were formed spontaneously at ambient temperature.

### 2.6. Color Measurement

Color variations in composite systems were determined using a colorimeter (Model LS173, Linshang Technology, Shenzhen, China) at 25 °C. The L* parameter represents lightness, ranging from 0 (pure black) to 100 (pure white), while the a* and b* values characterize chromaticity. The a* value ranges from −100 to +100, where negative values indicate greenness and positive values redness. Similarly, negative b* values correspond to blueness and positive values to yellowness [27].

### 2.7. Measurement of Physical Stability

The as-prepared composite systems were stored at ambient temperature (25 °C) for 3 days, and their storage stability was evaluated by measuring water separation rate at day 0, day 1 and day 3. Each sample was transferred into a 5 mL glass vial and heated in a boiling water bath at 100 °C for 15 min; thermal stability was quantified via the water separation rate after heating. For the freeze–thaw stability test, samples were frozen at −20 °C for 22 h and subsequently thawed at 25 °C for 2 h, followed by measurement of water separation rate. To assess centrifugal stability, the composite samples were centrifuged at 5000 rpm for 15 min before determining the water separation rate. HCl or NaOH solution (0.1–2.0 mol/L) was added to adjust the pH of the composite emulsions to 2.0, 4.0, 6.0, 8.0 and 10.0, and pH stability was evaluated based on the water separation rate. Various concentrations of NaCl solution were incorporated into the emulsions to reach final NaCl concentrations of 0, 50, 100, 150, 200 and 250 mM for ionic strength stability characterization via water separation measurement [28]. The water separation rate (%) was calculated as (mass of separated water/initial mass of sample) × 100%.

### 2.8. Rheological Characterization

All rheological measurements were performed on a TA Discovery rheometer (DHR, Shanghai, China) equipped with a 40 mm diameter parallel-plate geometry (DHR, TA Instruments, New Castle, DE, USA), and the measuring gap was set to 1 mm.

Steady-state flow tests were conducted over a shear rate range of 0.01–1000 s^−1^ for composite systems with varying NPGP concentrations (0.5 wt%, 1.0 wt%, 1.5 wt%, 2.0 wt%, 2.5 wt%). Amplitude sweep tests were carried out at a fixed frequency of 1 Hz within a strain range of 0.1–100%. Frequency sweeps were implemented at a constant strain of 0.5% located within the linear viscoelastic region (LVR), with angular frequency ranging from 0.1 rad/s to 100 rad/s. Thixotropy measurements were conducted via a three-stage shear rate ramp: shear rate was increased linearly from 0 to 100 s^−1^ and then decreased back to 0. The reversibility of viscosity variation was recorded to characterize structural breakdown and reformation under shear. Time sweep tests were performed at 25 °C with a fixed frequency of 1 Hz and constant strain of 0.5%. Storage modulus (G′) and loss modulus (G″) were continuously monitored for 600 s [29]. Creep-recovery tests were conducted using a constant stress selected from the LVR. The stress was applied for 300 s to record strain evolution during creep, followed by a 300 s recovery stage without applied stress to monitor strain restoration [30]. Large-amplitude oscillatory shear (LAOS) measurements were conducted at 1 Hz and 25 °C, with strain swept logarithmically from 0.1% to 1000%. Oscillatory strain, stress and shear rate signals were recorded at each strain amplitude to plot elastic and viscous Lissajous curves [31]. The elastic and viscous contributions at each strain amplitude were quantified using the MITLaos toolbox in MATLAB R2024a.

### 2.9. Texture

Textural attributes of all samples were evaluated via uniaxial compression and two-cycle texture profile analysis (TPA) using a TA-XT PLUS texture analyzer (Stable Micro Systems, Godalming, UK). A P/0.5 cylindrical probe was applied for uniaxial compression measurements. The instrumental parameters were set as follows: a pre-test speed of 1.0 mm/s, test speed of 0.1 mm/s, post-test speed of 1.0 mm/s, trigger force of 1.0 g, and penetration depth of 5 mm.

The identical P/0.5 probe was utilized for two-cycle TPA assays with a compression strain of 30% and a 5 s resting interval between two compression strokes. Hardness, springiness, gumminess, cohesiveness and resilience were quantified from the recorded force–time TPA curves [32].

### 2.10. Optical Microscopy Measurement

The morphological microstructures of samples were visualized under an optical microscope (MShot, Mingmei Optoelectronics Technology Co., Ltd., Guangzhou, China). A small portion of each sample was placed on a glass slide, and a coverslip was gently placed on top of the sample. The morphology and spatial arrangement of the emulsion droplets were observed at magnifications of 10 × 10 and 40 × 10.

### 2.11. 3D Printing and Stability Evaluation of Printed Structures

The samples were loaded into 50 mL cartridges fitted with a 0.6 mm diameter nozzle tip. A cylindrical digital model was designed for printing with a diameter of 20 mm, height of 17.5 mm, and infill density of 100%. The printing process was performed at ambient temperature with an extrusion rate of 50%. Printing accuracy and printing stability were evaluated at 25 °C (room temperature). Specifically, printing accuracy was quantified by measuring the dimensional deviation between printed specimens and the original digital model, while printing stability was assessed by comparing the deviation values of samples printed immediately (0 h) and those printed after a 12 h interval [33].

Subsequently, the printed samples were separately transferred into 5 mL glass vials. For thermal stability testing, samples were heated in a boiling water bath at 100 °C for 15 min; for freeze–thaw stability testing, samples were frozen at −20 °C for 22 h followed by thawing at 25 °C for 2 h. The water separation rate of treated samples was measured to characterize their post-printing thermal stability and freeze–thaw stability, respectively.

### 2.12. Statistical Analysis

All experiments were conducted with at least three biological replicates. All data were expressed as mean ± standard deviation. One-way analysis of variance (ANOVA) was performed using IBM SPSS Statistics 26 software (SPSS Inc., Chicago, IL, USA). Duncan’s multiple range test was adopted to analyze intergroup differences, where *p* < 0.05 indicated statistically significant differences. Origin 2024 software was utilized for plotting and statistical processing of experimental data.

## 3. Results and Discussion

### 3.1. Major Compositions of NPGP and Peanut Oil Bodies

Compositional analysis of NPGP presented a moisture content of 6.48 ± 0.12%, an ash content of 8.28 ± 0.34%, and an exceptionally high galacturonic acid content of 74.79 ± 0.05%, which verified that NPGP belonged to typical pectic polysaccharides, accompanied by a protein fraction of 3.28 ± 0.01%. As for peanut oil bodies, compositional detection yielded a crude fat content of 75.23 ± 0.72% and moisture content of 16.34 ± 0.15%, with phospholipid and protein contents reaching 0.33 ± 0.01% and 0.64 ± 0.07%, respectively. Such compositional profiles matched the inherent characteristics of peanut oil bodies, which are micro- to submicron organelles containing a lipid core surrounded by a membrane matrix composed of phospholipids and oil body-associated proteins [34].

### 3.2. Analysis of Appearance, Color and Physical Stability of Composite Systems

#### 3.2.1. Appearance and Color

**Figure 1:**
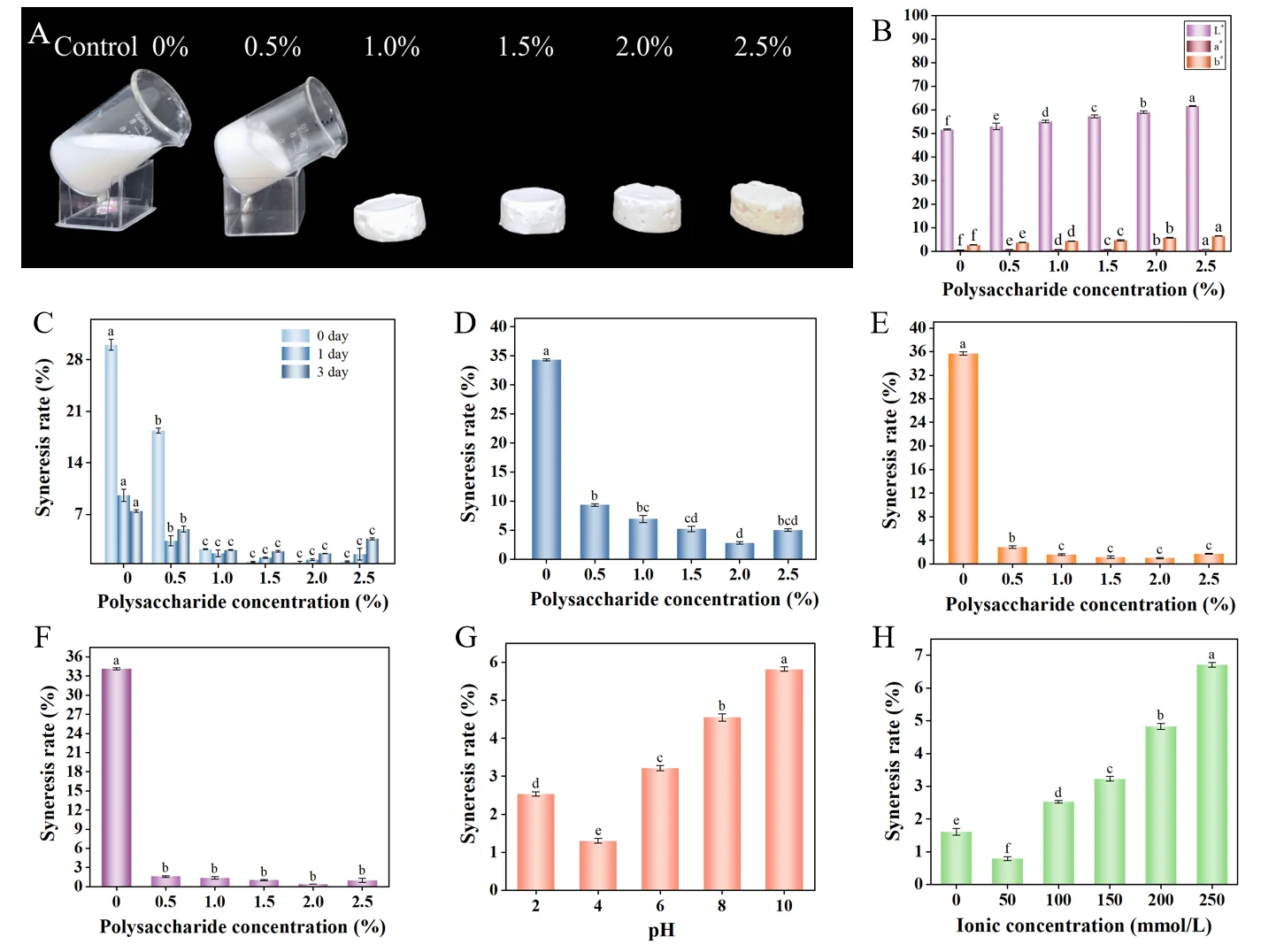
Characterization of (**A**) appearance, (**B**) color parameters, (**C**) storage stability, (**D**) thermal stability, (**E**) freeze–thaw stability, and (**F**) centrifugal stability of peanut oil body composite systems stabilized with different *Nicandra physalodes* (Linn.) Gaertn. polysaccharide (NPGP) concentrations; (**G**) pH stability and (**H**) ionic strength stability of peanut oil body emulsions stabilized by 2.0% NPGP. Different lowercase letters (a, b, c, d, e, f) indicate significant differences (*p* < 0.05).

The appearances of the peanut oil body emulsions stabilized with different NPGP concentrations are displayed in Figure 1A. The control group without NPGP addition and the sample supplemented with 0.5 wt% NPGP both appeared as free-flowing viscous liquids, whereas samples with NPGP concentrations ranging from 1.0% to 2.5% formed self-supporting solid-like gels. At an NPGP concentration of 2.5%, obvious rough textures emerged on the gel surface, and the color shifted from pure white to slightly creamy yellow. This phenomenon can be attributed to the excessive NPGP concentration, which impaired the homogeneity and transparency of the gel network. These results demonstrated that NPGP concentration acted as a critical factor governing the macroscopic morphology of the composite system.

Figure 1B illustrates the variations in color parameters of composite systems with different NPGP dosages. As the polysaccharide concentration increased from 0 to 2.5%, the lightness value L* of the systems showed an overall increasing tendency (*p* < 0.05). This trend was generally ascribed to improved dispersion of the emulsion induced by the polysaccharides, which intensified light scattering within the samples and thus raised the lightness [35]. The redness value a* remained close to 0 across all concentrations without significant differences, indicating a neutral tone along the red–green axis. The yellowness value b* increased moderately from 2.68 ± 0.06 to 6.51 ± 0.06 with elevated NPGP content, reflecting a mild intensification of yellow tint, which may stem from the intrinsic color of polysaccharides or intermolecular interactions between NPGP and oil body components. Overall, NPGP mainly regulated the color of peanut oil body composite systems by increasing lightness while moderately enhancing yellow tone, which provides a theoretical basis for optimizing the sensory quality of such emulsions.

#### 3.2.2. Characterization of Physical Stability

The physical stability of samples was comprehensively evaluated from multiple perspectives by measuring the water separation rate of various samples. NPGP concentration exerted significant effects on the storage, thermal, freeze–thaw and centrifugal stability of peanut oil body emulsions. For storage stability (Figure 1C), the water separation rate was determined on day 0, day 1 and day 3. The polysaccharide-free sample (0% NPGP) exhibited a high water separation rate of 30.06% on day 1, revealing the inherently poor stability of native oil body emulsions. The water separation rate decreased markedly as the polysaccharide concentration rose from 0.5% to 2.5%. At concentrations ≥ 1.0%, the water separation rate remained below 5%. This indicated that NPGP effectively slowed oil droplet aggregation and emulsion phase separation by elevating aqueous-phase viscosity and constructing a three-dimensional network, thereby greatly improving storage stability [36].

Thermal stability results are presented in Figure 1D. The emulsion without NPGP possessed a high thermal water separation rate of approximately 34.32%, demonstrating extreme susceptibility to thermal stress. The thermal water separation rate was significantly reduced at NPGP concentrations ≥ 0.5%, reaching 9.35% at 0.5%. When the concentration was raised to 2.0%, the thermal water separation rate dropped below 5%. Such outcomes confirmed that the polysaccharide network drastically enhanced thermal stability, as the continuous polysaccharide gel matrix increased system viscosity and gel strength to restrain the migration and aggregation of oil body particles under high temperatures [10].

Freeze–thaw stability is displayed in Figure 1E. The polysaccharide-free emulsion exhibited a freeze–thaw water separation rate of 35.69%, with its structure severely damaged during freeze–thaw cycles [11]. By contrast, the freeze–thaw water separation rate dropped below 5% at NPGP concentrations ≥ 0.5%, and the value varied slightly with further concentration increases, demonstrating that NPGP effectively protected the composite structure against freeze–thaw stress. Similar trends were observed for centrifugal stability (Figure 1F). The control emulsion without NPGP showed a centrifugal water separation rate of 34.13% and suffered severe phase separation under centrifugal force. After supplementation with ≥0.5% NPGP, the centrifugal water separation rate decreased to below 5% and remained steady within the range of 0.5–2.5%, which further verified the resistance of polysaccharide network structures to mechanical stress [37].

Collectively, the above results revealed that the water separation rate decreased remarkably and leveled off at NPGP concentrations ≥ 1.0%, and the composite system achieved a favorable and stable state at 2.0%. This tendency agrees with studies concerning soybean oil body emulsions stabilized by sodium alginate, which confirmed an obvious critical concentration threshold for polysaccharides [9]. Therefore, the stability of composite systems with varied pH and ionic strength was characterized at this optimal concentration. As illustrated in Figure 1G, the water separation rate of the composite system decreased first and then increased as pH rose. The minimum water separation rate (1.30%) was obtained at pH 4, corresponding to the optimal stability of the composite. This phenomenon was attributed to the partial protonation of carboxyl groups on NPGP chains under weak acidic conditions, which induced moderate chain contraction. The contracted polysaccharide chains could efficiently adsorb onto oil droplet surfaces to form compact protective films while retaining hydrated layers, thereby constructing a robust three-dimensional network [38]. In comparison, under strongly acidic conditions at pH 2, complete protonation of carboxyl groups further shrank polysaccharide chains yet greatly weakened their hydration capacity, raising the water separation rate. At strongly alkaline pH 10, complete dissociation of carboxyl groups caused excessive stretching of polysaccharide chains via strong electrostatic repulsion [39]. The resultant loose, thin interfacial films could not effectively stabilize oil droplets, thereby markedly increasing the water separation rate to 5.82%. These findings indicated that NPGP-stabilized emulsions were sensitive to pH fluctuations, with the optimal stability window located in the weak acidic range [18].

The water separation rate of the composite system showed a trend of first decreasing and then increasing with the increase in ionic strength in the range of 0–250 mmol/L (Figure 1H). The minimum water separation rate and optimal stability of the composite system were obtained at an ionic concentration of 50 mmol/L. A small amount of salt ions moderately shielded the electrostatic repulsion between polysaccharide molecular chains, promoting the formation of a denser and more ordered network structure and further enhancing the water retention capacity of the system. A further increase in ionic strength led to a significant rise in the water separation rate. This result was attributed to the strong electrostatic shielding effect of high-concentration ions on the repulsion between polysaccharide chains and oil droplet surfaces [40]. On the one hand, the aqueous network structure shrank and was destroyed; on the other hand, the electrostatic shielding induced the aggregation and coalescence of oil droplets, ultimately causing the overall structural instability of the emulsion and massive water precipitation.

### 3.3. Rheological Properties

**Figure 2:**
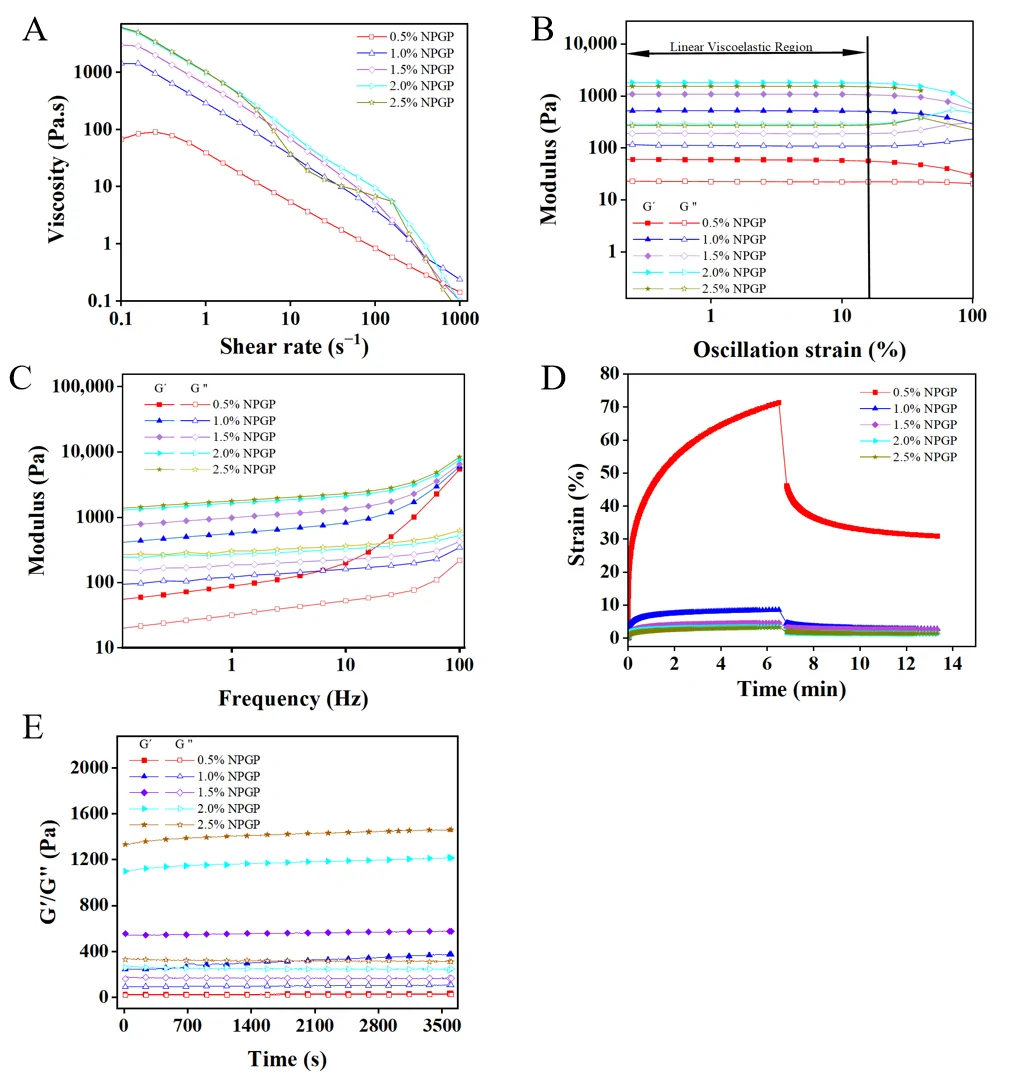
(**A**) Viscosity sweep, (**B**) strain sweep, (**C**) frequency sweep, (**D**) creep-recovery and (**E**) time sweep tests of peanut oil body emulsion composite systems stabilized with different concentrations of NPGP.

Rheological behavior acts as a critical factor governing the macroscopic stability and micro-texture of composite systems [41], and also serves as a critical indicator to judge their suitability for 3D printing. The viscosity sweep curves in Figure 2A reveal that the apparent viscosity of all samples declined with increasing shear rate, demonstrating the typical shear-thinning (pseudoplastic) behavior of the composites. As NPGP concentration rose from 0.5% to 2.5%, the initial viscosity at low shear rates increased markedly. The 2.5% NPGP sample reached an initial viscosity of 6230.81 Pa·s, while the value of the 0.5% group was only 1430.55 Pa·s; samples with higher polysaccharide concentrations maintained elevated viscosity across the entire tested shear rate range. This finding indicated that elevated NPGP dosage strengthened intermolecular interactions and oil–polysaccharide interfacial bonding, reinforced the three-dimensional network architecture, and accordingly improved overall viscosity and structural stability [42]. From the perspective of 3D printing, higher viscosity generally contributes to superior printing accuracy and better shape retention after extrusion [43].

Strain sweep measurements illustrated that the storage modulus (G′) and loss modulus (G″) of all samples remained constant within the LVR, and G′ was consistently higher than G″ for all groups, revealing elastic-dominated rheological performance. As presented in Figure 2B, the G′ and G″ curves lost parallelism once strain exceeded the critical threshold: G′ decreased continuously with growing strain, which signified irreversible breakdown of the internal network structure. G″ firstly increased to a local maximum before declining, corresponding to the characteristic weak strain overshoot phenomenon. A similar rheological trend was observed by Tang et al. [44] in the system of sodium caseinate interacting with locust bean gum and κ-carrageenan. Comparative analysis of modulus responses among samples with different NPGP concentrations showed that higher NPGP loading yielded a higher initial G′ plateau value, which increased from 61.13 Pa to 1527.24 Pa. A high storage modulus (G′) endows the system with strong resistance to external deformation. This phenomenon proved that increased polysaccharide content intensified the interactions between oil droplets and polysaccharide chains and boosted structural rigidity and elastic bearing capacity [45]. In terms of 3D printing, this means the extruded deposited layers possess sufficient mechanical strength to sustain the weight of upper printed layers and prevent collapse or deformation during multilayer stacking [46].

Frequency sweep tests can reveal the correlation between the composite system and its edible texture. As illustrated in Figure 2C, G′ was higher than G″ for all samples, and both moduli increased with rising frequency, revealing the solid-like elasticity-dominated rheological behavior of the composite systems [7]. This characteristic directly corresponds to the sensory attributes of gel-type foods, such as springiness and chewiness. Overall values of G′ and G″ rose and the gap between modulus curves widened as NPGP concentration increased from 0.5% to 2.5%. This indicated that higher polysaccharide concentrations reinforced the three-dimensional network and improved the elastic and mechanical strength of the system [15]. Moduli increased moderately at low frequencies but rose sharply at high frequencies, which agreed with the fact that polysaccharide chains barely relax under high frequency and thus maintain a more stable network structure.

Figure 2D presents the creep-recovery test results of composite systems with various NPGP concentrations. At the initial creep stage, strain of all samples increased rapidly at first and then grew at a slower rate over time, showing typical creep curve profiles. Strain values measured at identical time points decreased significantly with elevated NPGP loading, demonstrating that high-dose NPGP improved the creep resistance of the composites. The 0.5% NPGP sample exhibited the most severe creep and maximum deformation, while the 2.0% and 2.5% groups possessed similarly low strain values, suggesting that the reinforcing effect of NPGP nearly reached saturation at 2.0%. After stress removal, strain dropped rapidly and then entered a slow recovery phase, with a certain proportion of non-recoverable residual strain retained at the end. Creep-recovery behavior is closely related to the interlayer fusion performance and final morphology of 3D-printed products. Higher NPGP concentrations corresponded to a larger fraction of recoverable strain and smaller residual non-recoverable strain. As a filler, NPGP constructed interconnected networks with peanut oil bodies to restrict chain slippage, strengthen elastic response, suppress irreversible deformation, and ultimately improve the elastic recovery capacity of the system [47]. To a certain extent, such a property facilitates the extruded material to rapidly regain elastic support during 3D printing, maintaining uniform filament diameter and stable layer stacking while lowering the risk of interlayer collapse [48].

Time sweep tests were performed to monitor the formation and evolution of gel networks in composite systems consisting of peanut oil bodies and varying concentrations of NPGP. As illustrated in Figure 2E, all samples exhibited solid-like behavior (G′ > G″) throughout the testing period, with moduli remaining stable or slightly increasing over time. This verified that stable three-dimensional networks were formed without structural breakdown under continuous shear, demonstrating favorable time-dependent stability [28]. Both initial moduli and overall modulus levels increased systematically with higher NPGP concentrations. The incorporated polysaccharide enhanced crosslinking density within the continuous phase, which reflected the regulatory effect of NPGP concentration on network strength and structural stability. Such excellent temporal stability implies that the composite system can sustain consistent texture and rheological properties during storage, avoiding undesirable phenomena such as softening, syneresis or structural disintegration over time.

**Figure 3:**
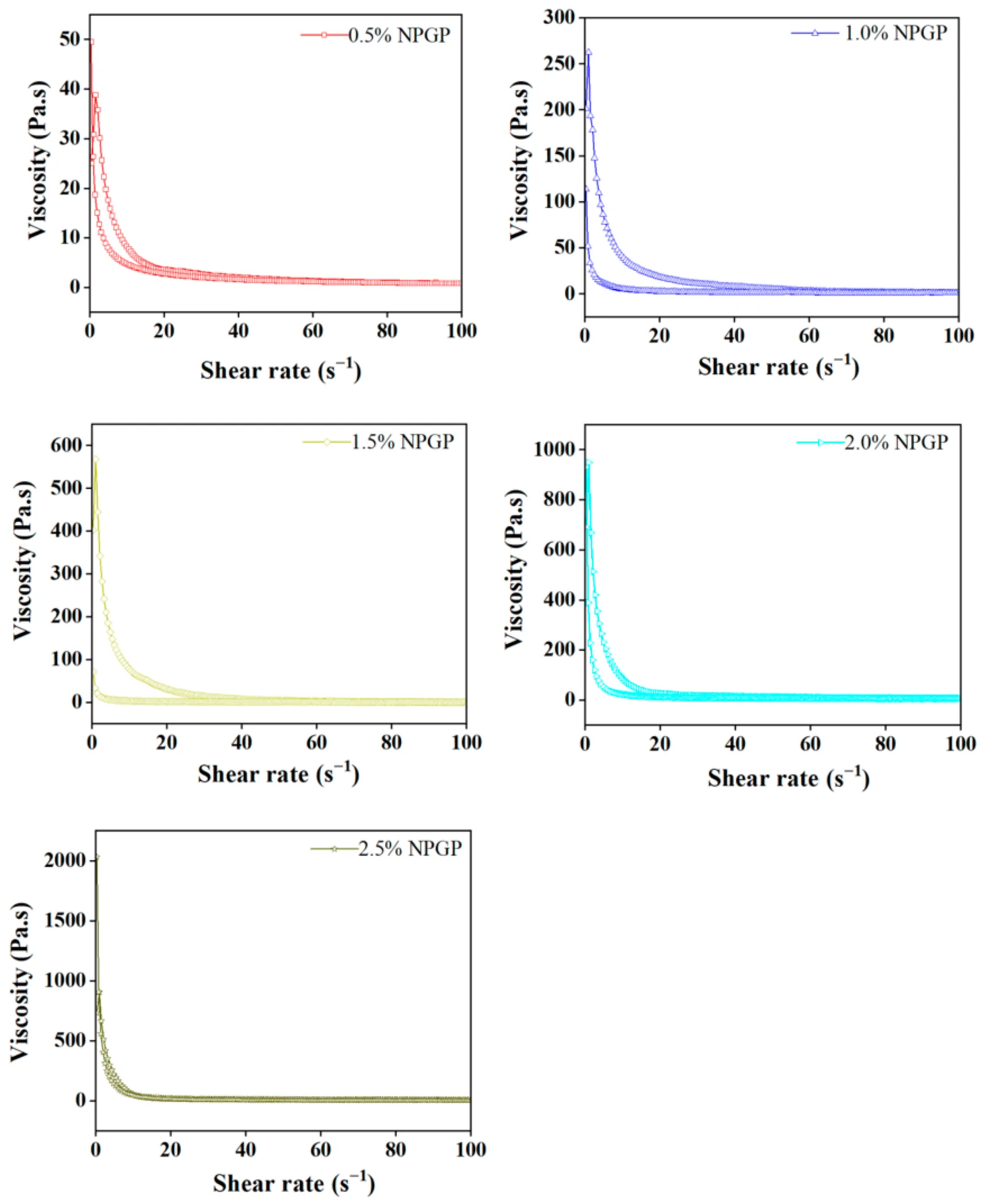
Thixotropy recovery ramps of peanut oil body emulsion composite systems stabilized with various NPGP concentrations.

Thixotropic properties of composite systems were characterized via continuous variable shear rate ramps, and the results are presented in Figure 3. The viscosity of all samples decreased remarkably with increasing shear rate, showing typical shear-thinning behavior and confirming that these composites belonged to pseudoplastic fluids. Apparent viscosity dropped sharply upon shear rate acceleration and recovered gradually as the shear rate decreased, generating distinct thixotropic hysteresis loops. The area enclosed by each hysteresis loop directly reflected the energy dissipation during shearing and the time lag between structural breakdown and reconstruction. As NPGP concentration increased from 0.5% to 2.5%, the initial viscosity at low shear rates rose significantly, which revealed that incorporated NPGP efficiently constructed intermolecular networks and greatly improved the static consistency of the system. Meanwhile, the area of thixotropic hysteresis loops expanded and the viscosity recovery ratio after shear cessation increased [45]. These phenomena demonstrated that higher NPGP concentrations strengthened the three-dimensional network and enhanced the reversibility of structural reconstruction after shear-induced damage. Such thixotropic properties play a vital role in determining the precision and accuracy of 3D-printed constructs. Systems with superior thixotropic performance exhibit better stability and storage capacity [49].

**Figure 4:**
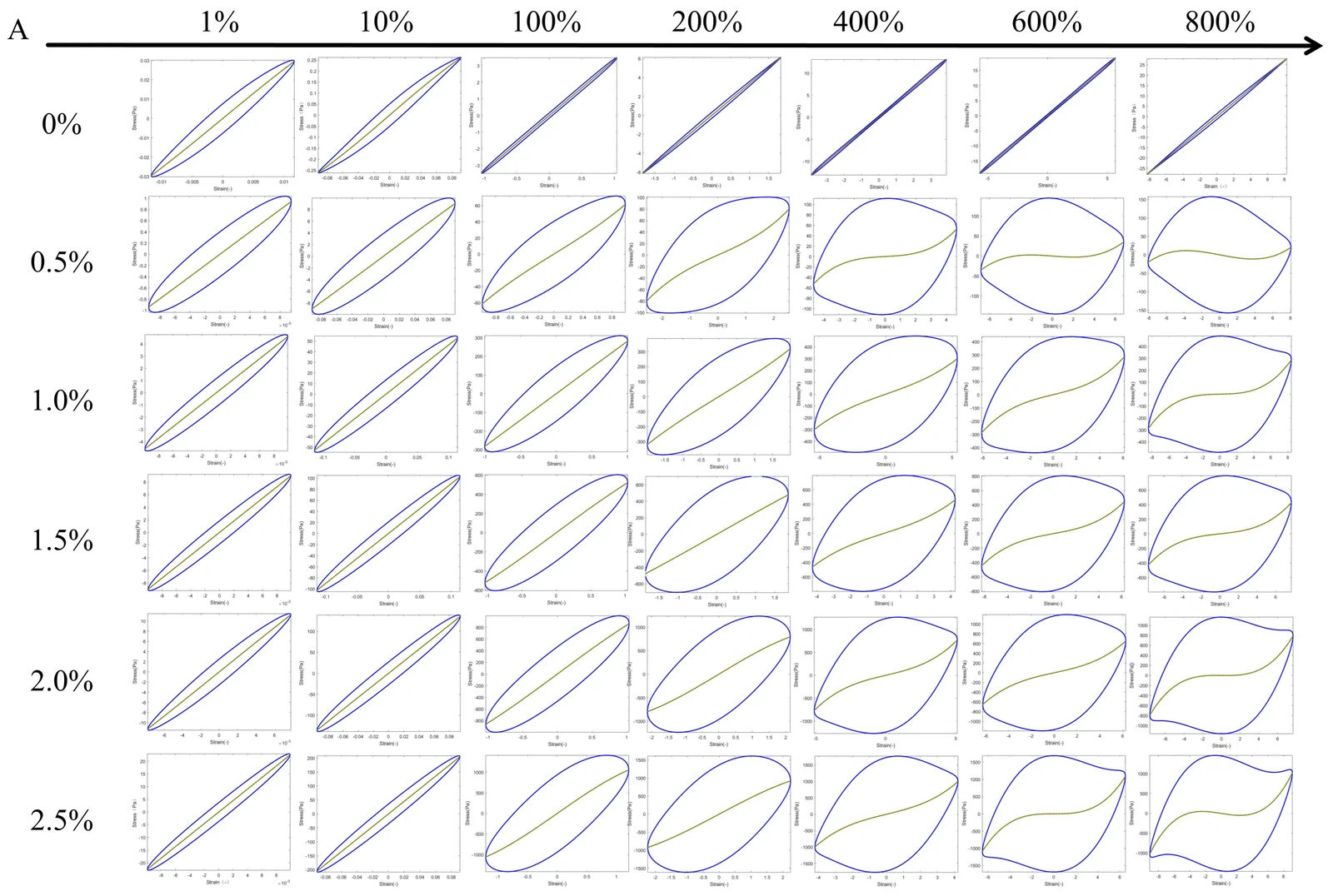

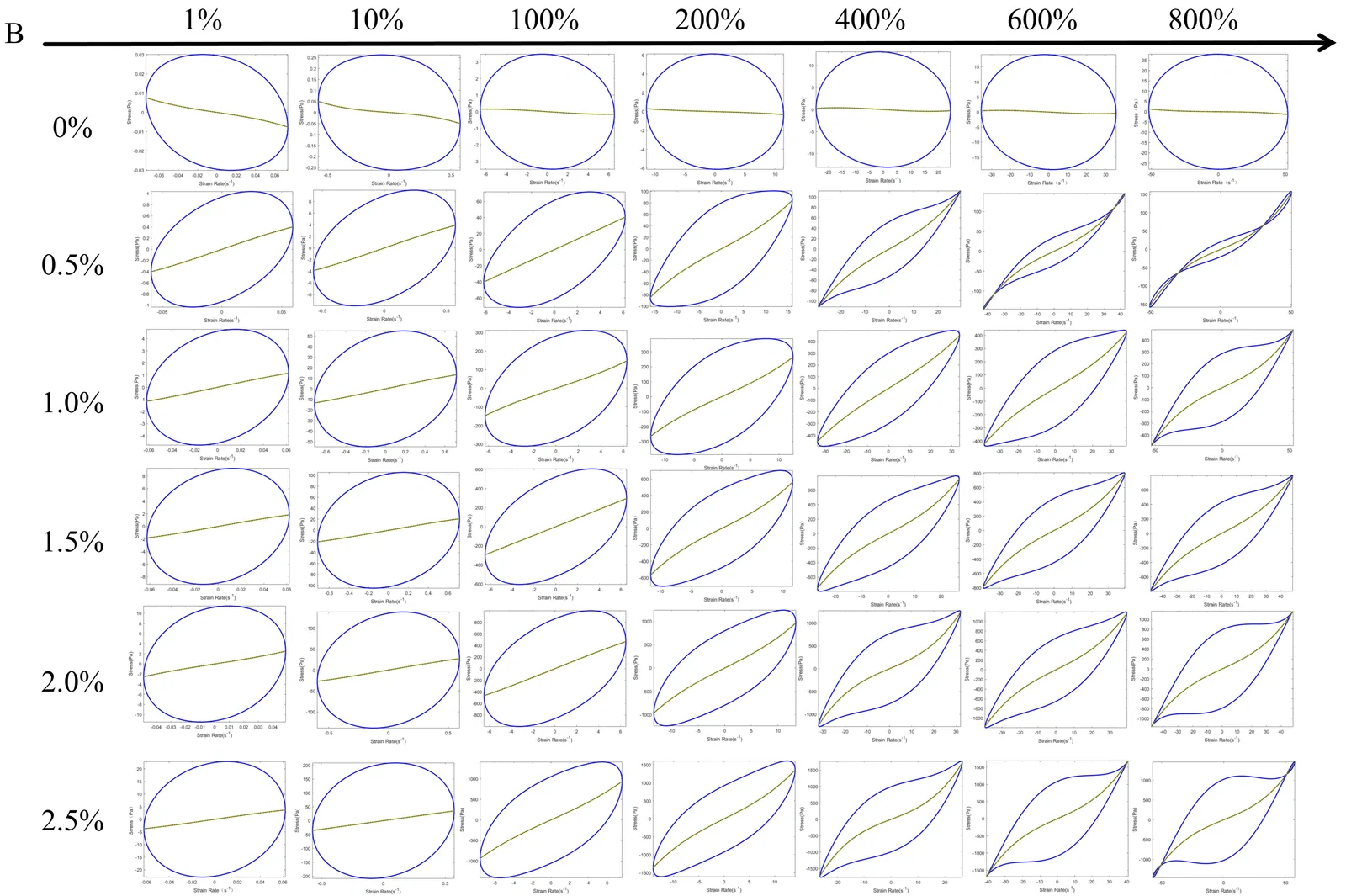
(**A**) Elastic Lissajous curves and (**B**) viscous Lissajous curves of peanut oil body emulsion composite systems stabilized with NPGP at concentrations ranging from 0 to 2.5%. The blue lines represent total stress, while the green lines correspond to elastic stress and viscous stress, respectively.

Lissajous curves were adopted to further investigate the nonlinear rheological behaviors of emulsion gels under large-amplitude oscillatory shear (LAOS). Figure 4 presents elastic and viscous Lissajous curves of emulsion gels measured at a fixed frequency of 1 Hz with various strain amplitudes (1%, 10%, 100%, 200%, 400%, 600% and 800%). In the linear regime at low strains (≤10%), the elastic Lissajous curves of the polysaccharide-free samples were nearly straight lines, characteristic of purely viscous fluids. After NPGP incorporation, the curves appeared as symmetric ellipses without distortion. As the polysaccharide concentration increased, the slope and linearity of the curves rose accordingly. This revealed that NPGP endowed the emulsion gels with solid-like elastic responses by forming physically crosslinked networks, and higher concentrations produced stronger elasticity within the linear viscoelastic plateau and denser network structures. A similar trend was reported in a study on pea protein–sodium alginate mixtures [50], in which the Lissajous curves of pea protein–sodium alginate systems displayed elastic features at low strain levels owing to their well-developed network structures.

In the intermediate strain-transition region (100–200%), Lissajous curves of low-concentration systems (0.5–1.0%) began to deform and evolved from symmetric ellipses to willow-leaf shapes, indicating the onset of shear-induced network breakdown and the transition into the nonlinear viscoelastic regime [51]. By contrast, curves of high-concentration samples (1.5–2.5%) retained favorable symmetry with markedly reduced distortion, which demonstrated that dense polysaccharide networks possessed superior shear resistance and an elevated critical breakdown strain.

Within the highly nonlinear regime at high strains (400–800%), Lissajous curves of low-concentration samples suffered severe distortion and nearly transformed into rectangular profiles, corresponding to typical shear softening and fluidization behaviors [48]. Moderate-concentration samples exhibited milder curve distortion and retained partial elastic contributions. Compared with all other concentrations, the 2.0% NPGP system displayed far slighter contour distortion, midline bending and asymmetric structural failure under large high-strain deformation.

In summary, NPGP enabled precise regulation of emulsion gel rheology via concentration-dependent three-dimensional network assembly. Increasing polysaccharide concentration effectively broadened the linear viscoelastic window and improved shear resistance. Comparable rheological trends have also been verified in emulsion gels composed of sesame oil bodies and various polysaccharides [52]. The incorporation of polysaccharides facilitates the formation of denser physically crosslinked networks, which is beneficial to achieving favorable 3D printability. The composite system with 2.0% NPGP possesses a well-developed elastic network, a wide linear viscoelastic range, and rapid network recovery after mechanical disruption. It exhibits comprehensive advantages for both ambient shelf storage and extrusion-based 3D printing, and thus holds promising prospects for fabricating structured edible plant-based foods via 3D printing.

### 3.4. Textural Characteristic

During the experiment, the composite system with 0.5% NPGP exhibited excessive fluidity and failed to form a stable gel structure, which did not meet the basic test requirements for texture profile analysis; thus, this group was excluded from measurements. As listed in Table 1, hardness characterizes the core capacity of composite systems to resist deformation under uniaxial compression and directly reflects their mechanical strength. Hardness increased significantly with the elevation of NPGP concentration and reached a maximum value of 36.15 ± 1.78 g at 2.0% NPGP. This phenomenon was attributed to the full hydration of polysaccharide chains, which constructed compact and homogeneous three-dimensional gel networks via hydrogen bonds, hydrophobic interactions and molecular entanglement. Such networks greatly improved the encapsulation and supporting capacity for the oil phase, thereby remarkably enhancing mechanical strength [53]. Firmness describes the overall structural strength of composite systems under biaxial compression and is closely correlated with shape retention stability. Its variation trend was highly consistent with hardness, showing a strong positive correlation. Meanwhile, springiness, cohesiveness and resilience of the composite systems all rose with increasing NPGP concentration and achieved optimal values at 2.0%. The obvious rise in cohesiveness indicated that the composite possessed stronger resistance to external damage and better structural integrity, which represented the internal viscoelastic strength of the matrix [54]. At this concentration, polysaccharide chains exhibited optimal hydration degree, crosslinking density and network homogeneity. The well-developed network efficiently encapsulated and immobilized peanut oil droplets, generating composite systems with balanced mechanical strength, flexibility and structural stability.

### 3.5. Optical Microscopy Observation

**Figure 5:**
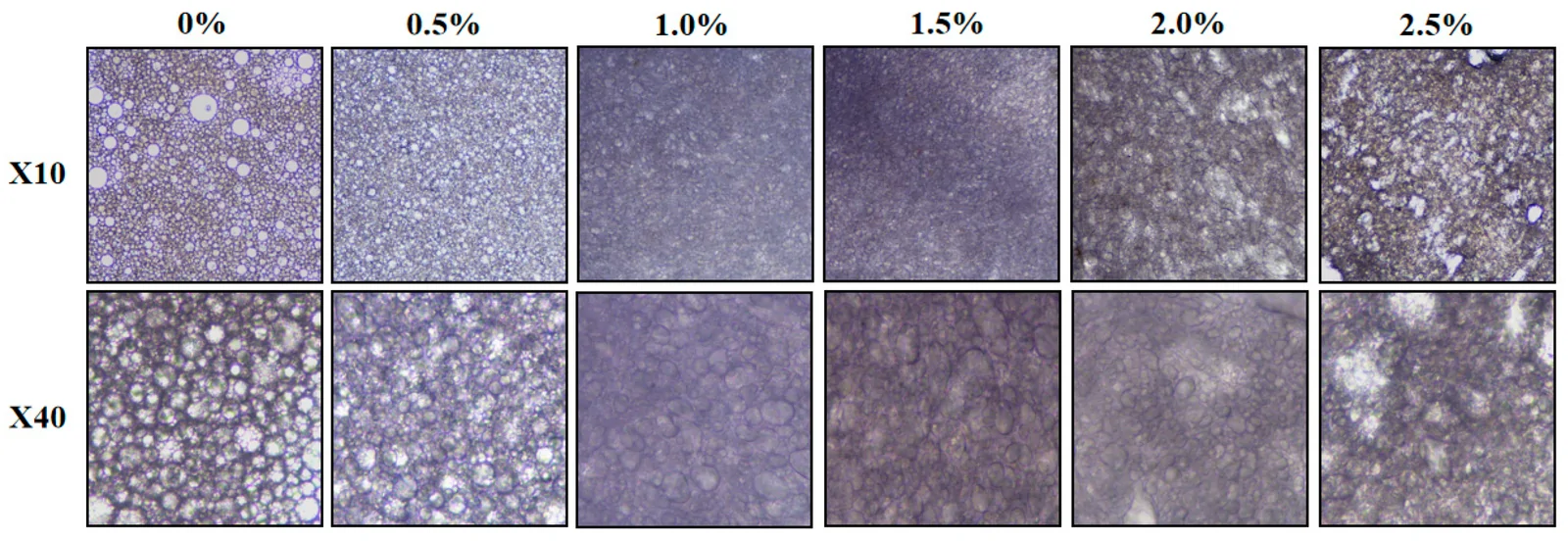
Optical micrographs of peanut oil body emulsion composite systems stabilized with NPGP at various concentrations. ×10 and ×40 represent magnifications of 10 × 10 and 10 × 40, respectively.

The optical micrographs displayed in Figure 5 visually reveal the regulatory effect of NPGP concentration on the microstructure of peanut oil body emulsions. In the polysaccharide-free control group, oil droplets exhibited large particle sizes and highly heterogeneous distribution, accompanied by severe aggregation and flocculation. When the NPGP concentration increased to 1.0%, the composite system was further homogenized, with most oil droplets existing as isolated individuals or tiny clusters. At 2.0% NPGP, oil droplets maintained a uniform distribution. A gel-like continuous phase could be observed in the background matrix, which effectively separated and encapsulated oil droplets. Nevertheless, further raising the polysaccharide concentration to 2.5% yielded inferior stabilizing performance compared with the 2.0% group. Excess polysaccharide constructed an overly dense continuous matrix, which may induce excessive network compaction or depletion flocculation, impairing system stability and reflected macroscopically by an elevated water separation rate.

Combined with macroscopic appearance photographs, the dense microstructures observed under optical microscopy were closely correlated with the overall morphology, stability and shape retention capacity of the samples. Formulations containing 1.0–2.5% NPGP formed self-supporting crosslinked polysaccharide gels that did not collapse upon inversion. This demonstrated that NPGP not only optimized oil droplet dispersion, but also thickened the aqueous phase and built continuous polysaccharide networks to strengthen matrix rigidity and water-binding capacity. The synergistic effects greatly improved macroscopic stability and structural integrity [55], laying a favorable foundation for further processing and 3D printing applications of the composite.

### 3.6. Analysis of 3D Printing Performance and Stability of Composite Systems

#### 3.6.1. 3D Printability

The extrudability and printability of semi-solid materials for 3D printing are governed by their rheological properties, including viscosity, shear-thinning behavior, elastic performance and yield stress [56]. The composite systems possessed suitable flowability and shape stability required for 3D printing, which preliminarily verified their feasibility as 3D printing inks. On this basis, further 3D printing tests were carried out to systematically investigate the forming stability and shape retention capacity during printing, so as to evaluate their comprehensive applicability under practical printing conditions.

**Figure 6:**
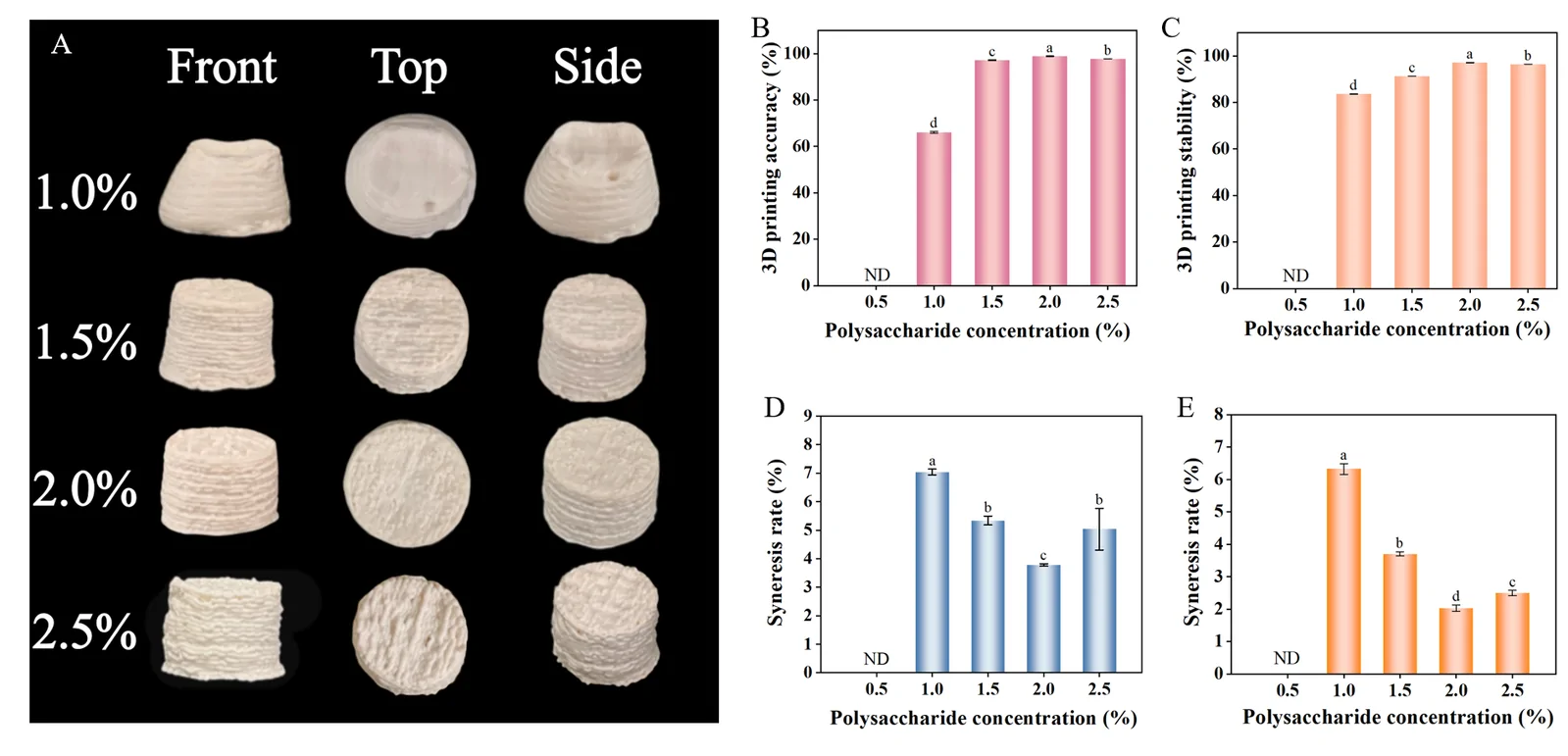
3D printing performances of peanut oil body emulsion composite systems stabilized with various concentrations of NPGP: (**A**) macroscopic appearance, (**B**) printing accuracy, and (**C**) printing storage stability; post-printing characterization of (**D**) thermal stability and (**E**) freeze–thaw stability. Different lowercase letters (a, b, c, d) indicate significant differences (*p* < 0.05).

During 3D printing, the composite with 0.5% NPGP exhibited excessive fluidity and failed to support its own weight, making it impossible to form intact printed structures; thus this group was excluded from testing. The macroscopic appearance of 3D-printed samples prepared with composite systems of varying NPGP concentrations is presented in Figure 6A, which reveals that NPGP concentration exerted a remarkable influence on printability. At 1.0% NPGP, severe collapse occurred in printed samples, with obvious pores and depressions on the top surface. Disordered layer lines on the side failed to maintain a regular cylindrical profile. With increasing NPGP concentration, the morphology of printed samples was improved, and the pre-designed cylindrical shape could be roughly retained. At 2.0% NPGP, the printed samples possessed regular shapes and distinct edges. Uniform and dense layer lines were observed on both top and side surfaces without obvious collapse, delamination or deformation, demonstrating outstanding printability.

3D printing accuracy reflects the matching degree of dimensions and morphology between printed samples and pre-designed models, and the corresponding results are displayed in Figure 6B. The printing accuracy of the 1.0% NPGP group was only 66.06%, accompanied by prominent dimensional and morphological deviations. When NPGP concentration increased to 1.5%, the printing accuracy sharply rose to approximately 97.14%. Further elevation of polysaccharide concentration enabled printed samples to fit the pre-set models more closely in both size and shape. Storage stability quantitatively characterizes the deformation resistance of stacked printed samples after several hours of placement, and the relevant data are shown in Figure 6C. The storage stability of the 1.0% NPGP sample was 83.61%, indicating that the structure was prone to deformation after 12 h of storage. Storage stability increased significantly with higher NPGP concentrations and reached a maximum value of 97.11% at 2.0%, suggesting that the printed structure maintained excellent stability even after 12 h of standing.

#### 3.6.2. Stability Characterization of Post-Printing Products

To evaluate the quality retention capacity of 3D-printed products, thermal stability (Figure 6D) and freeze–thaw stability (Figure 6E) were characterized by measuring the water separation rate of the printed composites, which quantitatively reflected their structural stability as well as their water- and oil-holding capacity. As NPGP concentration increased from 1.0% to 2.5%, post-printing thermal and freeze–thaw stability exhibited highly consistent trends: the water separation rate decreased markedly at first and then plateaued. The minimum water separation rate was achieved at 2.0% NPGP. It can be concluded that 2.0% NPGP was the optimal concentration to maximize the stability of printed composite systems. At this concentration, dense and homogeneous network structures were formed, which not only guaranteed high printing accuracy and favorable printability, but also endowed the printed products with outstanding thermal and freeze–thaw resistance.

## 4. Conclusions

This study investigated the stabilization performance of peanut oil body emulsions stabilized with different concentrations of NPGP. The results demonstrated that NPGP effectively improved the stability of peanut oil body emulsions, optimized oil droplet dispersion, and endowed the composite systems with favorable viscoelasticity and shape-forming capacity. The composite system with 2.0% NPGP exhibited optimal comprehensive physical properties. Further rheological tests revealed that the composite possessed suitable yield stress, typical shear-thinning behavior and favorable structural recovery capacity, which satisfied the requirements of extrudability and shape retention during 3D printing. 3D-printed constructs prepared from the composite achieved high printing accuracy and structural integrity, indicating that NPGP-stabilized peanut oil body emulsions possess great application potential as printable food matrices. This work not only broadens the utilization of NPGP natural resources, but also provides a novel and efficient strategy for stabilizing peanut oil body emulsions with natural NPGP, offering theoretical support for the functional development of 3D-printed food materials.

## Figures and Tables

**Table 1:** Textural properties of peanut oil body emulsion composite systems stabilized with different concentrations of NPGP.

Samples	Hardness (g)	Firmness (N)	Springiness	Cohesiveness	Resilience
1.0%	8.29 ± 1.34 c	0.11 ± 0.00 c	34.79 ± 1.49 b	0.43 ± 0.01 b	51.39 ± 0.02 c
1.5%	32.94 ± 1.06 b	0.26 ± 0.00 b	75.28 ± 1.17 a	0.80 ± 0.01 a	64.28 ± 1.09 a
2.0%	36.15 ± 1.78 a	0.30 ± 0.01 a	75.94 ± 1.87 a	0.81 ± 0.01 a	64.38 ± 1.51 a
2.5%	35.08 ± 0.72 ab	0.29 ± 0.01 a	75.44 ± 1.79 a	0.78 ± 0.05 a	62.12 ± 1.28 b

Note: Different lowercase letters indicate significant differences in textural parameters among samples (*p* < 0.05).

## Data Availability

The original contributions presented in the study are included in the article; further inquiries can be directed to the corresponding author.

## References

[1] Nikiforidis C.V. (2019). Structure and functions of oleosomes (oil bodies). Adv. Colloid Interface Sci..

[2] Hao J., Zhang F., Decker E.A., Sun B., Xu D. (2024). Study on the interfacial properties of rice bran oil bodies affecting their physicochemical properties, stability and adsorption behavior at the oil-water interface for the design of precisely emulsified systems. Food Hydrocoll..

[3] Hao J., Li X., Wang Q., Lv W., Zhang W., Xu D. (2022). Recent developments and prospects in the extraction, composition, stability, food applications, and in vitro digestion of plant oil bodies. J. Am. Oil Chem. Soc..

[4] Shi Z., Chen Z., Meng Z. (2023). Study on oil body emulsion gels stabilized by composited polysaccharides through microgel particles compaction and natural gelation. Food Hydrocoll..

[5] Şen A., Acevedo-Fani A., Dave A., Ye A., Husny J., Singh H. (2024). Plant oil bodies and their membrane components: New natural materials for food applications. Crit. Rev. Food Sci. Nutr..

[6] Mohamed S.A.A., El-Sakhawy M., El-Sakhawy M.A.M. (2020). Polysaccharides, Protein and Lipid -Based Natural Edible Films in Food Packaging: A Review. Carbohydr. Polym..

[7] Shi Z., Xu W., Geng M., Chen Z., Meng Z. (2023). Oil body-based one-step multiple phases and hybrid emulsion gels stabilized by sunflower wax and CMC: Application and optimization in 3D printing. Food Hydrocoll..

[8] Zhu T., Wang S., Yan D., Zhang L., Guo X., Chen F. (2025). Preparation, interaction, and digestion of peanut oil body-based emulsion gels with xanthan gum and gallic acid. Food Hydrocoll..

[9] Su C., Feng Y., Ye J., Zhang Y., Gao Z., Zhao M., Yang N., Nishinari K., Fang Y. (2018). Effect of sodium alginate on the stability of natural soybean oil body emulsions. RSC Adv..

[10] Farooq S., Ahmad M.I., Ali U., Zhang H. (2024). Fabrication of curcumin-loaded oleogels using camellia oil bodies and gum arabic/chitosan coatings for controlled release applications. Int. J. Biol. Macromol..

[11] Liao Y., Sun Y., Wang Z., Zhong M., Li R., Yan S., Qi B., Li Y. (2022). Structure, rheology, and functionality of emulsion-filled gels: Effect of various oil body concentrations and interfacial compositions. Food Chem. X.

[12] Han H., Zhao L., Liu X., Guo A., Li X. (2020). Effect of water bath-assisted water extraction on physical and chemical properties of soybean oil body emulsion. Food Sci. Nutr..

[13] Wu L., Yue Q., Kang M., Zhong M., Qi B., Li Y. (2022). Stabilization of soybean and peanut oil bodies using apple pectin under acidic conditions. Colloids Surf. A Physicochem. Eng. Asp..

[14] Wu N.-N., Huang X., Yang X.-Q., Guo J., Zheng E.-L., Yin S.-W., Zhu J.-H., Qi J.-R., He X.-T., Zhang J.-B. (2012). Stabilization of soybean oil body emulsions using ι-carrageenan: Effects of salt, thermal treatment and freeze-thaw cycling. Food Hydrocoll..

[15] Hashemi B., Assadpour E., Zhang F., Jafari S.M. (2023). A comparative study of the impacts of preparation techniques on the rheological and textural characteristics of emulsion gels (emulgels). Adv. Colloid Interface Sci..

[16] Fu K., Wang H., Pan T., Cai Z., Yang Z., Liu D., Wang W. (2024). Gel-forming polysaccharides of traditional gel-like foods: Sources, structure, gelling mechanism, and advanced applications. Food Res. Int..

[17] Zeng Q., Deng L., Wang R., Liu H., Chen J., Dai T., Wang Y., Liu C., Liang R. (2025). The mechanism of emulsion stabilization by protein-free pectin from *Nicandra physaloides* (Linn.) *Gaertn* seeds: Part I. the effect of esterification degree. Food Hydrocoll..

[18] Guo C., Li X., Gong T., Yang X., Wang G., Yang X., Guo Y. (2021). Gelation of *Nicandra physalodes* (Linn.) Gaertn. polysaccharide induced by calcium hydroxide: A novel potential pectin source. Food Hydrocoll..

[19] Liu H., Deng L., Dai T., Chen J., Liu W., Liu C., Chen M., Liang R. (2022). Emulsifying and emulsion stabilization mechanism of pectin from *Nicandra physaloides* (Linn.) *Gaertn* seeds: Comparison with apple and citrus pectin. Food Hydrocoll..

[20] Εkonomou S.Ι., Hadnađev M., Gioxari A., Abosede O.R., Soe S., Stratakos A.C. (2024). Advancing dysphagia-oriented multi-ingredient meal development: Optimising hydrocolloid incorporation in 3D printed nutritious meals. Food Hydrocoll..

[21] Ghimire S., Gamonpilas C., Umar M., Anal A.K. (2025). Production of 3D-printed meat analogues using pea, fava, and mung bean proteins: A comparison study. Food Struct..

[22] Wang Y.-Y., Chen F.-S., Lv D.-Y., Jia L.-A., Guo X.-F. (2025). Modulating the interfacial structure of peanut oil body emulsions through different extraction methods: Implications for curcumin delivery. LWT.

[23] (2016). National Food Safety Standard—Determination of Moisture Content in Foods.

[24] (2016). National Food Safety Standard—Determination of Ash in Foods.

[25] (2016). National Food Safety Standard—Determination of Protein in Foods.

[26] (2008). Inspection of Grain and Oils—Determination of Phosphatide Content.

[27] Bibat M.A.D., Ang M.J., Eun J.-B. (2022). Impact of replacing pork backfat with rapeseed oleosomes–Natural pre-emulsified oil–On technological properties of meat model systems. Meat Sci..

[28] Lv D., Chen F., Yang X., Yin L., Yu J., Chen Z. (2024). Ficus awkeotsang Makino pectin in acidic environments: Insights into pectin structure, gelation behavior, and gel properties. Carbohydr. Polym..

[29] Lv D., Chen F., Yang X., Yin L. (2025). Composite gels prepared from pea protein isolate and *Ficus awkeotsang* makino pectin: Spontaneous gelation behavior, microstructures, and formation mechanism. Food Chem..

[30] Farooq S., Ahmad M.I., Zhang Y., Zhang H. (2023). Impact of interfacial layer number and Schiff base cross-linking on the microstructure, rheological properties and digestive lipolysis of plant-derived oil bodies-based oleogels. Food Hydrocoll..

[31] Li Q., Xu M., Xie J., Su E., Wan Z., Sagis L.M., Yang X. (2021). Large amplitude oscillatory shear (LAOS) for nonlinear rheological behavior of heterogeneous emulsion gels made from natural supramolecular gelators. Food Res. Int..

[32] Ji Y., McClements D.J., Luo M., Wang Q., Li M., Rashid A., Wang W. (2026). Fabrication of edible inks for 3D food printing: High internal phase Pickering emulsions stabilized by polysaccharide-polyphenol particles. Food Chem..

[33] Qiao G., Chen X.-W., Luo X.-Y., Zhang L.-S., Liu X. (2026). Switchable thermo-triggered and 3D-printable soy protein soft emulsion gels as fat analogs for plant-based meat. Food Hydrocoll..

[34] Zhou L., Chen F., Hao L., Du Y., Liu C. (2019). Peanut Oil Body Composition and Stability. J. Food Sci..

[35] Hu X., McClements D.J. (2022). Construction of plant-based adipose tissue using high internal phase emulsions and emulsion gels. Innov. Food Sci. Emerg. Technol..

[36] Xu W., Yin Y., Yue M., Sun H., Kang M., Luo D., Shah B.R., Ge Y. (2024). Construction of highly stable Pickering emulsion systems based on konjac glucomannan and xanthan gum/lysozyme nanoparticles under pasteurization. Food Chem. X.

[37] Chen C., Tian Q., Yi J., Hu X., Du M., Jiang Y. (2024). Anise Essential Oil Nano-Emulsion Prepared with *Nicandra physalodes* (Linn.) Gaertn. Seed Pectin: Optimization, Characterization, Stability, and Bioactivity. SSRN.

[38] Yang X., Guo C., Yang Y., Yuan K., Yang X., Guo Y. (2022). Rheological and gelling properties of *Nicandra physalodes* (Linn.) Gaertn. pectin in acidic media. Food Chem..

[39] Lopez C., Rabesona H., Novales B., Weber M., Anton M. (2023). Walnut (*Juglans regia* L.) kernel oil bodies recovered by aqueous extraction for utilization as ingredient in food emulsions: Exploration of their microstructure, composition and the effects of homogenization, pH, and salt ions on their physical stability. Food Res. Int..

[40] Sriprablom J., Luangpituksa P., Wongkongkatep J., Pongtharangkul T., Suphantharika M. (2019). Influence of pH and ionic strength on the physical and rheological properties and stability of whey protein stabilized o/w emulsions containing xanthan gum. J. Food Eng..

[41] Li D., Zhao Y., Wang X., Tang H., Wu N., Wu F., Yu D., Elfalleh W. (2020). Effects of (+)-catechin on a rice bran protein oil-in-water emulsion: Droplet size, zeta-potential, emulsifying properties, and rheological behavior. Food Hydrocoll..

[42] Shi F., Chang Y., Shen J., Chen G., Xue C. (2023). A comparative investigation of anionic polysaccharides (sulfated fucan, ι-carrageenan, κ-carrageenan, and alginate) on the fabrication, stability, rheology, and digestion of multilayer emulsion. Food Hydrocoll..

[43] Bom S., Ribeiro R., Ribeiro H.M., Santos C., Marto J. (2022). On the progress of hydrogel-based 3D printing: Correlating rheological properties with printing behaviour. Int. J. Pharm..

[44] Tang M.-X., Lei Y.-C., Wang Y., Li D., Wang L.-J. (2021). Rheological and structural properties of sodium caseinate as influenced by locust bean gum and κ-carrageenan. Food Hydrocoll..

[45] Li S., Li P., Wang J., Lu Y., Chen Y., Zhao Z., Jiang J., Cheng X., Bi L. (2024). Characterization and stability of low-oil emulsion gels with newly shaped droplets stabilized by camellia saponin and k-carrageenan. Food Hydrocoll..

[46] Zheng Z., Zhang M., Liu Z. (2021). Investigation on evaluating the printable height and dimensional stability of food extrusion-based 3D printed foods. J. Food Eng..

[47] Li J., Guo C., Cai S., Yi J., Zhou L. (2023). Fabrication of anthocyanin–rich W1/O/W2 emulsion gels based on pectin–GDL complexes: 3D printing performance. Food Res. Int..

[48] Lin Q., Shang M., Li X., Sang S., Chen L., Long J., Jiao A., Ji H., Qiu C., Jin Z. (2024). Rheology and 3D printing characteristics of heat-inducible pea protein-carrageenan-glycyrrhizic acid emulsions as edible inks. Food Hydrocoll..

[49] Liu Y., Zhang Y., Cai L., Zeng Q., Wang P. (2024). Protein and protein-polysaccharide composites-based 3D printing: The properties, roles and opportunities in future functional foods. Int. J. Biol. Macromol..

[50] Yoon W.B., Jung H., Oyinloye T.M. (2024). Synergistic Effects of Pea Protein on the Viscoelastic Properties of Sodium Alginate Gels: Findings from Fourier Transform Infrared and Large-Amplitude Oscillatory Shear Analysis. Processes.

[51] Schreuders F., Sagis L., Bodnár I., Erni P., Boom R., van der Goot A. (2021). Small and large oscillatory shear properties of concentrated proteins. Food Hydrocoll..

[52] Yang Y., Yang R., Wang W., Fang Y., Zhao L. (2026). Polysaccharides reinforced sesame oil body interface as 3D printing inks for dysphagia food: Focusing on structural characteristics and rheological properties. Food Chem. X.

[53] Samakradhamrongthai R.S., Maneechot S., Wangpankhajorn P., Jannu T., Renaldi G. (2022). Polydextrose and guar gum as a fat substitute in rice cookies and its physical, textural, and sensory properties. Food Chem. Adv..

[54] Farooq S., Ahmad M.I., Zhang Y., Chen M., Zhang H. (2023). Preparation, characterization and digestive mechanism of plant-derived oil bodies-based oleogels structured by chitosan and vanillin. Food Hydrocoll..

[55] Chen Q., Ji H., Wang Z., Wang Y., Wang X., Chen Z. (2025). Effects of different charged polysaccharides on the gelation properties and in vitro digestibility of potato protein gel: Insight into underlying mechanisms. Food Hydrocoll..

[56] Johannesson J., Pathare M.M., Johansson M., Bergström C.A., Teleki A. (2023). Synergistic stabilization of emulsion gel by nanoparticles and surfactant enables 3D printing of lipid-rich solid oral dosage forms. J. Colloid Interface Sci..

